# A new model to predict major bleeding in patients with atrial fibrillation using warfarin or direct oral anticoagulants

**DOI:** 10.1371/journal.pone.0203599

**Published:** 2018-09-10

**Authors:** J’Neka S. Claxton, Richard F. MacLehose, Pamela L. Lutsey, Faye L. Norby, Lin Y. Chen, Wesley T. O’Neal, Alanna M. Chamberlain, Lindsay G. S. Bengtson, Alvaro Alonso

**Affiliations:** 1 Department of Epidemiology, Rollins School of Public Health, Emory University, Atlanta, GA, United States of America; 2 Division of Epidemiology and Community Health, School of Public Health, University of Minnesota, Minneapolis, MN, United States of America; 3 Cardiovascular Division, Department of Medicine, University of Minnesota Medical School, Minneapolis, MN, United States of America; 4 Division of Cardiology, Department of Medicine, School of Medicine, Emory University, Atlanta, GA, United States of America; 5 Department of Health Sciences Research, Mayo Clinic, Rochester, MN, United States of America; 6 Health Economics and Outcomes Research, Life Sciences, Optum, Eden Prairie, MN, United States of America; Maastricht University Medical Center, NETHERLANDS

## Abstract

**Background:**

No scores presently exist to predict bleeding in atrial fibrillation (AF) populations using direct oral anticoagulants (DOACs). We used data from two independent healthcare claims databases to develop and validate a predictive model of major bleeding in a contemporary AF population.

**Methods:**

Patients with non-valvular AF initiating oral anticoagulation were identified in the MarketScan databases from 2007–2014. Using Cox regression models in 1000 bootstrapped samples, we developed a model that selected variables predicting major bleeding in the first year after anticoagulant initiation. The final model was validated in patients with non-valvular AF in the Optum Clinformatics database in the period 2009–2015. The discriminative ability of existing bleeding scores were individually evaluated and compared with the new bleeding model termed Anticoagulation-specific Bleeding Score (ABS) in both MarketScan and Optum.

**Results:**

Among 119,083 patients with AF initiating oral anticoagulation in the derivation cohort, 4,030 experienced a bleeding event. The variable selection model identified 15 variables (including individual type of oral anticoagulant) associated with major bleeding. Discrimination of the model was modest [c-statistic 0.68, 95% confidence interval (CI) 0.67–0.69]. The model was subsequently applied to 81,285 AF patients in the validation data set (3,238 bleeding events), showing similar discrimination (c-statistic 0.68, 95% CI 0.67–0.69). In both cohorts, the predictive performance of the ABS was better than the existing models for bleeding prediction in AF.

**Conclusions:**

We developed a model that uses administrative healthcare data for the identification of AF patients at higher risk of bleeding after initiation of oral anticoagulation, taking into account the lower bleeding risk in DOAC compared to warfarin users.

## Introduction

Atrial fibrillation (AF), a common cardiac arrhythmia, profoundly increases the risk of morbidity and mortality from stroke and several other cardiovascular diseases.[1, 2] Anticoagulation therapy reduces the risk of stroke,[3] thus the American College of Cardiology/American Heart Association/Heart Rhythm Society Guideline for the Management of Patients With Atrial Fibrillation recommends oral anticoagulants (OACs) for patients with a moderate to high risk of stroke.[4] Anticoagulation, however, carries an increased risk of bleeding complications.[5] Therefore, physicians and patients must balance the risk of stroke against the risk of serious hemorrhage when determining whether or not to initiate anticoagulation therapy.

Currently, there are several risk stratification schemes available to quantify bleeding risk in AF patients,[6–9] however, all were developed in individuals on warfarin. Historically, vitamin K antagonists (VKA; mostly warfarin in the United States) were the only available OACs for use in patients with AF. However, recently the Food and Drug Administration approved four direct oral anticoagulants (DOACs) for the prevention of ischemic stroke and cardioembolic complications. In randomized clinical trials these anticoagulants–dabigatran, rivaroxaban, apixaban, and edoxaban–demonstrated non-inferiority to warfarin for stroke prevention and were associated with lower rates of hemorrhage.[10–14] DOACs have different pharmacological profiles and fewer drug-drug interactions than warfarin,[4] and therefore current bleeding risk scores, developed in patients on warfarin-only, may not have the same utility in patients using DOACs. These schemes also do not provide an inclusive assessment of the risks and benefits of different types of OACs given the patient’s characteristics. Therefore, there is a need for a contemporary classification system to guide the decision between specific OACs based on the individual patient characteristics associated with bleeding risk.

Using data from large healthcare utilization databases in the US, we developed a model for the prediction of major bleeding in AF patients who initiated OAC therapy with either VKA or non-VKA anticoagulants. We validated the model in an external sample of patients contained in a separate large US healthcare utilization database. We then compared the performance of our model to four major bleeding risk scores (HEMORR_2_HAGES,[8] HAS-BLED,[7] ATRIA,[9] and ORBIT [6]) in both the derivation and validation data.

## Methods

### Study population

This study uses health care utilization data from two large US databases, MarketScan and Optum Clinformatics, to construct a predictive model of major hemorrhage associated with oral anticoagulation use in AF patients. Data from MarketScan (2007–2014) was used to develop the risk equation and the validation of the model was performed in the data from Optum Clinformatics (2009–2015). A brief description of each database is provided below. For each database, the analysis was restricted to individuals with available medical and outpatient pharmacy claims data, with at least six months of continuous enrollment prior to the first prescription of an oral anticoagulant, and a history of non-valvular AF. History of AF was defined as at least one inpatient or two outpatient claims at least 7 days but less than 1 year apart with the International Classification of Disease Ninth Revision Clinical Modification (ICD-9-CM) code 427.31 or 427.32 in any position. Individuals with any inpatient ICD-9-CM codes for mitral stenosis or mitral valve disorders were excluded. With this exclusion, however, our definition of non-valvular AF does not include patients with mitral valve regurgitation, mild mitral valve stenosis, or any valvular diseases that would be considered as having non-valvular AF according to AF guidelines. [4, 15] Additionally, patients were required to have a first prescription for an oral anticoagulant (warfarin or DOAC) after their initial AF diagnosis. Participants with an oral anticoagulant prescription prior to AF diagnosis were presumed to use these agents for other conditions (e.g., venous thromboembolism). The Institutional Review Board at Emory University reviewed and approved this study.

#### Derivation cohort

We used claims data from the Truven Health MarketScan® Commercial Claims and Encounters Database and the Medicare Supplemental and Coordination of Benefits Database (Truven Health Analytics Inc., Ann Arbor, MI) for the period January 1, 2007 to December 31, 2014. The MarketScan Commercial Database includes health insurance claims and enrollment data collected from large US employers and health plans that provide private healthcare coverage for employees, their spouses and dependents. The Medicare Supplemental Database includes claims from individuals and their dependents with Medicare supplemental plans. Each database links patient enrollment data with medical and outpatient prescription drug claims and encounter data, providing individual clinical use, expenditure, and outcomes across inpatient and outpatient services and outpatient pharmacy services.

#### Validation cohort

We used claims data from Clinformatics^®^ Data Mart, a product of Optum (Eden Prairie, MN), for the period January 1, 2009 to September 30, 2015. The database includes Commercial Claims Data and Managed Medicare data. The Commercial Claims Database includes health insurance claims and enrollment data collected from a diverse group of health plans in the United States. The Medicare Database includes claims from individuals enrolled in a Managed Medicare health plan. Each database links individual-specific medical (inpatient and outpatient) and pharmacy claims with patient enrollment data.

### Oral anticoagulant use

In both datasets, outpatient pharmaceutical claims were identified for each eligible individual. Each claim includes information on the National Drug Code (NDC), the prescription fill date, and the number of days supplied. All prescriptions for oral anticoagulants (warfarin, dabigatran, rivaroxaban, and apixaban) from January 1, 2007 to December 31, 2014 (MarketScan) and January 1, 2009 to September 30, 2015 (Optum Clinformatics) were identified. Edoxaban was not included primarily because it received FDA approval (January 2015) outside the study period in the derivation (MarketScan) cohort and there were few users in the validation (Optum Clinformatics) cohort. Patients were categorized according to their first anticoagulant prescription after their AF diagnosis, disregarding if they discontinued or switched to a different oral anticoagulant. All DOAC prescriptions were included independently of the dosage strength, which assumes that patients were prescribed their correct dose given their characteristics. The validity of warfarin claims in administrative databases is excellent with a positive predictive value (PPV) of 99%;[16] no information is available on the validity of claims for DOAC prescription.

### Definition of major bleeding event

The main outcome variable was hospitalization for a major bleeding event (intracranial, gastrointestinal, or other) that occurred after OAC initiation. Intracranial hemorrhage was defined by the presence of ICD-9-CM codes 430 (subarachnoid hemorrhage) and 431 (intracerebral hemorrhage) as the primary diagnosis in an inpatient claim. Validation studies have demonstrated that these codes have a PPV of >90%.[17] Gastrointestinal bleeding was defined in accordance with an algorithm developed by Cunningham et al.[18] The algorithm considers presence of bleeding-related ICD-9-CM codes in inpatient claims as primary diagnoses, presence of transfusion codes (hospital revenue code indicating transfusion/cross-matching for transfusion), and presence/absence of trauma-related codes to exclude bleeding events resulting from major trauma. The PPV for this algorithm was 86%,[18] which compares favorably to other published algorithms.[19] Other bleeding events also were defined based on the Cunningham et al. algorithm.[18] In this category we included hemopericardium, hemoperitoneum, hemarthrosis, hematuria, vaginal bleeding, epistaxis, hemorrhage from throat, hemoptysis, hemophthalmos, hematoma of soft tissue, hemorrhage complicating a procedure, and unspecified hemorrhage. The PPVs range from 80% to 100%.[18]

### Candidate predictors

We identified 35 candidate predictors of major bleeding from literature and other prediction models (i.e. HEMORR_2_HAGES,[8] HAS-BLED,[7] ATRIA,[9] and ORBIT [6]). These variables were defined based on validated algorithms when available,[18, 20] using ICD-9-CM codes from inpatient, outpatient, and pharmacy claims prior to or at the time of OAC initiation. These variables included comorbidities and pharmacy claims, in addition to age, sex, and variables included in prior prediction models.[6–9] The following comorbidities were evaluated: ischemic stroke, heart failure, coronary artery disease, hypertension, diabetes mellitus, peripheral artery disease, liver disease, kidney disease, chronic pulmonary disease, prior history of bleeding (i.e., gastrointestinal bleeding, intracranial bleeding, or other bleeding), anemia, coagulopathy, cancer, dementia, depression, myocardial infarction, and alcoholism. A patient was considered to have a comorbidity if they had an inpatient or outpatient claim including any of the relevant diagnostic ICD-9-CM codes in any position prior to or at the time of OAC initiation; a list of ICD-9-codes used to define each variable is included in S1 Table. The following prescription medications were defined using pharmacy claims, based on NDCs: angiotensin converting enzyme inhibitors, calcium channel blockers, beta blockers, angiotensin receptor blockers, diuretics, lipid lowering medications, insulin, sulfonylureas, metformin, thiazolidinediones, dipeptidyl peptidase-4 inhibitors, other oral antidiabetics, antiplatelets, type I antiarrhythmics (i.e., quinidine and flecainide), and type III (i.e., amiodarone and dronedarone), other antiarrhythmics, digoxin, and non-steroidal anti-inflammatory drugs.

### Statistical analysis

#### Derivation of the predictive model

In this analysis, patients were categorized according to their first prescribed OAC after AF diagnosis. Follow-up started at the date of the first OAC and continued until a major bleeding hospitalization occurred, December 31, 2014, or patient health plan disenrollment, whichever occurred earlier.

To identify potential predictors of severe hemorrhage, we constructed four drug-specific multivariable Cox proportional hazards models with time to major bleeding hospitalization as the outcome. The candidate predictors, described in the previous section, were included in each multivariable model. We generated 1000 bootstrap samples from the MarketScan cohort and ran Cox proportional hazards models with backward selection of the 35 candidate predictor variables in each of the 1000 samples, with a p> 0.05 for exclusion. Variables included in at least 60% of bootstrap samples in one or more drug-specific models were selected for inclusion in the final prediction model.[21] Next, we tested for interactions between the oral anticoagulants and the covariates selected from the bootstrap samples and selected those with p<0.05. We then assessed the performance of the model using the C-statistic to estimate the discrimination of the model. Calibration was qualitatively assessed by plotting observed risk within deciles of predicted risks.[22] Finally, we compared the discriminative ability of our new model (termed Anticoagulation-specific Bleeding Score (ABS)) with four other risk prediction scores, HEMORR_2_HAGES, HAS-BLED, ATRIA, and ORBIT (summarized in S2 Table), using the C-statistic. Each score was reconstructed according to the definitions used in their respective derivation cohorts, using variables that were defined prior to or at the time of OAC initiation in the MarketScan databases. Several score components were not available in the database and thus contributed zero points to the given score; cells for missing components are shaded in S2 Table.

#### External model validation

The prediction model developed in the derivation cohort was applied in the Optum Clinformatics cohort to estimate the 1-year risk of a major bleeding event. As in the derivation analysis, model performance was assessed using the C-statistic and plotting observed risk within deciles of predicted risks. We also evaluated the discrimination of HEMORR_2_HAGES, HAS-BLED, ATRIA, and ORBIT in the validation cohort. Each score was calculated in the Optum Clinformatics database as described above. All statistical analyses were performed with SAS 9.4 (SAS Institute, Cary, NC).

## Results

### Derivation cohort

For the period of January 1, 2007 to December 31, 2014, the MarketScan databases included 1,150,742 patients with a diagnosis of non-valvular AF. Among those patients, we identified 119,083 individuals initiating OAC after their AF diagnosis with at least 180 days of enrollment data prior to OAC initiation. Table 1 presents demographic and clinical characteristics of these patients at the time of OAC initiation. The majority of patients (69%) were warfarin initiators, followed by rivaroxaban (13%), dabigatran (12%), and apixaban (6%). Approximately six percent of oral anticoagulant initiators switched to a different anticoagulant during the first year. Overall, warfarin users were older, equally or more likely to be women, had higher risk scores for stroke (CHADS_2_ and CHA_2_DS_2_-VASc) and bleeding (HAS-BLED, ATRIA, HEMORR_2_HAGES, and ORBIT), and higher prevalence of comorbidities compared to DOAC initiators.

**Table 1:** Characteristics of patients with atrial fibrillation according to initial prescribed anticoagulant in the derivation (MarketScan, 2007–2014) and validation (Optum Clinformatics, 2009–2015) cohorts.

	Derivation Cohort (MarketScan)				Validation Cohort (Optum Clinformatics)			
	Warfarin	Dabigatran	Rivaroxaban	Apixaban	Warfarin	Dabigatran	Rivaroxaban	Apixaban
N	82,205	14,611	15,695	6,572	49,894	9,088	14,043	8,260
**Demographics**								
Age, years	71.0 (12.7)	68.2 (12.7)	68.1 (12.7)	69.3 (12.6)	73.9 (10.4)	70.4 (11.3)	71.6 (11.2)	73.7 (10.8)
Women, %	41.1	37.0	39.6	41.2	44.2	40.1	42.6	47.3
**Existing stroke risk scores**								
CHADS_2_	2.5 (1.6)	2.2 (1.5)	2.1 (1.5)	2.2 (1.5)	3.0 (1.5)	2.5 (1.5)	2.6 (1.5)	2.8 (1.5)
CHA_2_DS_2_-VASc	3.8 (2.1)	3.4 (2.1)	3.3 (2.1)	3.5 (2)	4.6 (2.0)	3.9 (2.0)	4.1 (2.1)	4.4 (2.0)
**Existing bleeding risk scores**								
HAS-BLED	2.3 (1.3)	2.2 (1.3)	2.1 (1.3)	2.2 (1.3)	2.8 (1.3)	2.5 (1.3)	2.7 (1.3)	2.8 (1.3)
ATRIA	3.1 (2.5)	2.6 (2.2)	2.6 (2.3)	2.7 (2.4)	4.1 (2.8)	3.2 (2.5)	3.5 (2.7)	3.9 (2.8)
HEMORR_2_HAGES*	2.6 (1.8)	2.3 (1.7)	2.3 (1.7)	2.4 (1.7)	3.3 (1.9)	2.7 (1.8)	3.0 (1.8)	3.2 (1.9)
ORBIT	1.7 (1.7)	1.4 (1.6)	1.4 (1.6)	1.4 (1.6)	2.2 (1.8)	1.7 (1.7)	1.9 (1.8)	2.1 (1.8)
**Prevalent disease, %**								
Heart failure	36.3	29.4	27.5	29.8	45.5	33.9	35.1	38.8
Coronary heart disease	42.1	38.9	36.7	38.0	47.3	41.2	41.9	44.0
Hypertension	77.2	79.6	79.8	82.3	89.0	87.9	87.8	89.3
Diabetes	32.8	29.7	28.7	29.4	39.9	34.4	35.8	37.0
Stroke	28.2	24.3	22.6	23.0	33.4	27.8	28.8	32.7
Peripheral artery disease	16.6	14.3	14.1	14.2	25.7	18.9	23.1	24.2
Kidney disease	14.7	9.0	10.0	12.6	25.9	15.8	19.4	23.8
Liver disease	6.1	6.3	6.7	6.5	8.1	7.3	9.4	9.5
Prior gastrointestinal bleed	10.5	10.1	9.4	8.7	12.4	10.8	12.1	13.4
Prior other bleed	12.6	12.3	11.9	11.2	16.0	13.8	16.9	17.2
Prior intracranial bleed	1.7	1.0	1.1	1.3	2.1	1.1	1.4	1.9

Numbers correspond to mean (SD) and percentages

* Not all variables were available in the datasets to calculate this score (See Supplementary S2 Table), thus this score was not reconstructed as originally defined.

**Fig 1:**
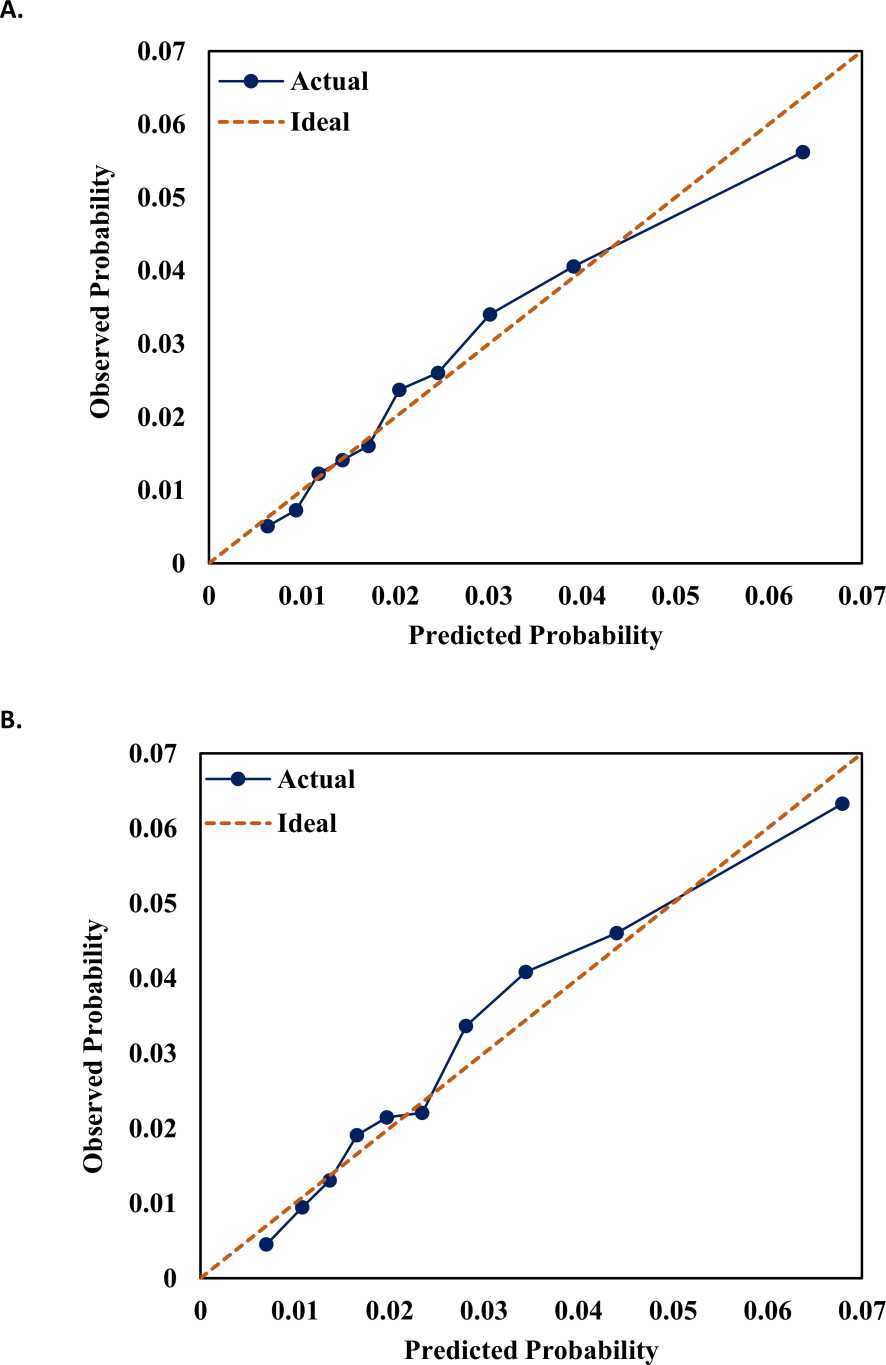
Calibration of final model in derivation and validation cohorts. Calibration curve relating observed and predicted bleeding rates across deciles of risk in **A.** Derivation Cohort (MarketScan) **B.** Validation Cohort (Optum Clinformatics). The 45 degree dashed line indicates perfect fit.

In the derivation cohort, over a mean (median) follow-up of 21 (15) months, we identified 4,030 hospitalizations with a bleeding event (intracranial, gastrointestinal, or other) in the primary position. Of these events, there were 3,355 major bleeds among warfarin users, 335 among dabigatran users, 289 among rivaroxaban users and 51 among apixaban users. The corresponding bleeding rates were 1.97, 1.37, 2.12, and 1.43 bleeds per 100 patient-years (Table 2). The following 14 variables were selected in at least 60% of the MarketScan bootstrap samples and one or more drug-specific models: age, kidney disease, prior bleeding, stroke, anemia, history of cancer, antiplatelet use, antiarrhythmic use, chronic pulmonary disease, heart failure, coronary artery disease, diuretics use, diabetes mellitus, and male sex (S3 Table). We added type of OAC to the model, resulting in a 15-variable model. We also found significant interactions of OAC use with age, kidney disease, and sex. These three interaction terms were initially considered for inclusion in the final model and were tested in both the derivation and validation cohorts. The discrimination of the model with interaction terms in the derivation cohort was fair (C-statistic = 0.68, 95%CI 0.67–0.69.) and showed good calibration (S1A Fig). However, in the validation sample the model with interaction experienced fair discrimination (C-statistic = 0.68, 95%CI 0.67–0.69), but poor calibration, with the model underpredicting risk (S1B Fig). Therefore, in efforts to improve the calibration in the validation sample, we decided to proceed with a model that did not include any interactions. In the derivation sample, this model had fair discrimination (C-statistic = 0.68, 95%CI 0.67–0.69) and good calibration (Fig 1A). Table 3 shows the beta coefficients, hazard ratios and 95% CIs from the individual variables in the derivation sample. The formula to calculate the 1-year bleeding risk based on these variables is included as a table footnote. We also provide an Excel-based calculator that computes 1-year risk estimates of major hemorrhage hospitalization for each anticoagulant, using the baseline risk function and the coefficients from the Cox model in the derivation sample (S1 File). In addition, Table 4 shows the discrimination of each of the existing models (HEMORR_2_HAGES, HAS-BLED, ATRIA, and ORBIT) and the newly developed model in the derivation cohort. In the derivation cohort, ABS displayed the best discrimination compared to the other models.

**Table 2:** Observed bleeding rates in patients with non-valvular AF initiating oral anticoagulation per 100 person-years in the derivation (MarketScan, 2007–2014) and validation (Optum Clinformatics, 2009–2015) cohorts.

**Derivation Cohort** **(MarketScan)**				
** **	**Warfarin**	**Dabigatran**	**Rivaroxaban**	**Apixaban**
**No. events**	3355	335	289	51
**Person-years**	170,797	24,517	13,660	3,576
**Incidence Rate (95% CI)**	1.97 (1.9, 2.04)	1.37 (1.23, 1.52)	2.12 (1.88, 2.36)	1.43 (1.04, 1.82)
**Validation Cohort** **(Optum Clinformatics)**				
** **	**Warfarin**	**Dabigatran**	**Rivaroxaban**	**Apixaban**
**No. events**	2420	282	411	125
**Person-years**	102,978	19,909	18,880	7,729
**Incidence Rate (95% CI)**	2.35 (2.26, 2.44)	1.42 (1.25, 1.58)	2.18 (1.97, 2.39)	1.62 (1.33, 1.9)

**Table 3:** Final model coefficients and hazard ratios in the derivation cohort, MarketScan, 2007–2014.

VARIABLE	BETA COEFFICIENTS	HR (95%CI)
Age, per 1 year	0.02306	1.02 (1.02,1.03)
Kidney disease	0.29958	1.35 (1.24,1.46)
Chronic obstructive pulmonary disease	0.19215	1.21 (1.13,1.30)
Prior bleeding event	0.23529	1.27 (1.18,1.36)
Anemia	0.32257	1.38 (1.29,1.48)
Heart failure	0.21811	1.24 (1.16,1.33)
Antiplatelet therapy	0.22599	1.25 (1.16,1.35)
Diuretics	0.15944	1.17 (1.10,1.26)
Diabetes mellitus	0.21110	1.24 (1.16,1.32)
History of Cancer	0.16955	1.19 (1.10,1.28)
Antiarrhythmic drugs	-0.28572	0.75 (0.66,0.85)
Ischemic stroke	0.13743	1.15 (1.07,1.23)
Coronary artery disease	0.10269	1.11 (1.03,1.19)
Sex (male)	-0.04775	0.95 (0.89,1.02)
DOAC (vs warfarin)		
Dabigatran	-0.30127	0.74 (0.66,0.83)
Rivaroxaban	0.01299	1.01 (0.90,1.15)
Apixaban	-0.52426	0.59 (0.45,0.78)

The 1-year risk of bleeding can be calculated as 1 - (0.98101)**Exp[0.02306*(Age -70.1736) + 0.29958*(Kidney Disease -0.13244) + 0.19215*(Chronic Obstructive Pulmonary Disease -0.31286)+ 0.23529*(Prior Bleed-0.21338) +0.32257*(Anemia -0.24892) + 0.21811*(Heart Failure-0.33899)+ 0.22599*(Antiplatelet-0.16341) + 0.15944*(Diuretics-0.4518) + 0.2111*(Diabetes Mellitus-0.31686) + 0.16806*(Cancer-0.16955) - 0.28572*(Antiarrhythmic -0.11919) + 0.13743*(Ischemic stroke -0.26681) + 0.10269*(Coronary Artery Disease -0.40768) - 0.04775*(Male Sex-0.59637) - 0.30127*(Dabigatran) + 0.01299*(Rivaroxaban) - 0.52426*(Apixaban)]

**Table 4:** Model discrimination [c-statistic (95% confidence interval)] by derivation and validation cohorts.

*Score*	Derivation cohort (MarketScan)	Validation cohort (Optum Clinformatics)
**Anticoagulation-specific Bleeding Score (New model)**	0.68 (0.67, 0.69)	0.68 (0.67, 0.69)
**HAS-BLED score**	0.64 (0.63, 0.66)	0.63 (0.62, 0.65)
**ATRIA score**	0.65 (0.64, 0.66)	0.65 (0.64, 0.66)
**HEMORR_2_HAGES score**	0.65 (0.64, 0.66)	0.64 (0.63, 0.65)
**ORBIT score**	0.65 (0.64, 0.66)	0.65 (0.64, 0.66)

### Validation cohort

Subsequently, we applied our bleeding risk score (ABS) to the validation sample. Using the same inclusion criteria as in MarketScan, the validation sample included 81,285 patients with non-valvular AF and at least 180 days of enrollment before OAC initiation, for which the majority were warfarin users. Similar to the derivation sample, warfarin users in Optum Clinformatics were older and had higher risk scores for stroke and bleeding, as well as higher prevalence of comorbidities compared to those initiating DOACs (Table 1). Seven percent of oral anticoagulant initiators switched to a different anticoagulant during the first year. We identified 3,238 major bleeds (warfarin: 2,420; dabigatran: 282; rivaroxaban: 411; apixaban: 125) with a mean (median) follow-up time of 22 (17) months. Bleeding rates were higher in the validation cohort (Table 2). Discrimination of the new model was similar to that observed in the derivation cohort (C-statistic = 0.68, 95%CI 0.67–0.69), and higher than those of the other bleeding scores (Table 4). The model also demonstrated good to moderate discrimination when assessed in each anticoagulant individually from the validation cohort (S4 Table). Fig 1B shows the calibration of the new model in the validation cohort, which was acceptable and similar to the calibration in the derivation cohort. The predictive ability of the model was poorer for patients ≥75 years of age (S5 Table), with adequate calibration in both datasets (S2 Fig).

## Discussion

Using a large administrative healthcare database, we identified 15 variables associated with major bleeding and developed a model that estimates bleeding risk of patients on either warfarin or DOACs. In the derivation data set, the discrimination of the model was modest. We validated the model in a second data set, showing similar model discrimination and adequate calibration. Our model had slightly better predictive ability than existing ones. The model’s performance was decreased in those ≥75 years.

Patients with AF eligible for anticoagulation are often not receiving it because of clinicians’ fear of adverse bleeding outcomes.[23] Therefore, quantifying an individual’s risk of major hemorrhage is a vital component to effective anticoagulation and overall AF management. There have been several risk stratification schemes developed to assess bleeding risk, however, our model has several advantages over current schemes.[6–9] First, ABS provides a comprehensive assessment of the bleeding risk in a contemporary population of patients with AF initiating OAC. With the introduction of DOACs, there is need for a model that provides clinicians and patients with more quantitative information to evaluate the risk-benefit ratio of anticoagulation therapy, which goes beyond the binary aspect of a positive or negative recommendation. Developed in a database that includes patients on either warfarin or DOACs, our model describes bleeding risk by type of OAC initiated and takes into account the described lower risk of bleeding in DOACs compared to warfarin. In contrast, current scores (HEMORR_2_HAGES,[8] HAS-BLED,[7] ATRIA,[9] and ORBIT [6]) were developed in warfarin only patients and only dichotomize patients, categorizing them as low risk or high risk of bleeding (possibly requiring closer observation), but do not provide finer estimation of risk. Second, the Excel-based calculator we developed using the predictive model can be used by clinicians to facilitate the decision-making process. Although the large number of variables in the model may seem an obstacle to its use, our score could be easily implemented into clinical settings because of the use of claim-based diagnostic codes, which are readily available in electronic medical record systems. For example, predicted bleeding risk based on our model could be automatically calculated using available prior clinical information and shown to providers as part of a patient dashboard or clinical decision support system. Third, because there are no laboratory requirements to determine components of the score, there is limited concern for lack of data availability, compared to the HEMORR_2_HAGES and HAS-BLED scores, which require information on variables such as genetic factors and labile INR. Additionally, ‘labile INR’ is not a relevant measure in those taking DOACs or those who are first time initiators of OACs. Fourth, the ABS was developed and validated in datasets with a large number of major bleeding events, allowing the identification of a larger number of predictors and a more precise inference than prior scores. The databases also include an extensive amount of clinical information on comorbidities and prescription medications that were considered risk factors for major bleeding. Fifth, the model was validated in a usual-practice setting using an external population of patients obtained from a separate US commercial claims database.

This study has several limitations intrinsic to administrative claims data that should be considered. Foremost, our definition of major bleeding and candidate predictors were based on ICD-9-CM diagnostic codes. As such, the predictive ability of our model depends on the ability to ascertain accurately both outcome and covariates from administrative data. These variables, however, were defined according to validated algorithms with PPVs between 80–100%. Second, our assessment of predictors of major bleeding are dependent on risk factors available in the database. Variables including potentially relevant information, such as genetic susceptibility, were not available. This limitation also extends to our assessments of the bleeding risk score, HEMORR_2_HAGES. Caution should therefore be taken when interpreting results for this score due to the lack of availability, in our data, of variables needed to reconstruct this score as originally defined. Third, in this analysis, a patient’s follow-up did not end if there were changes in anticoagulation therapy or concerns with medication compliance or discontinuation following initiation. Approximately 6–7% of patients in each database changed their anticoagulation therapy during the first year of follow-up, however these changes were not expected to affect the overall risk of bleeding after initiation of the first anticoagulant. Fourth, DOAC prescriptions were included independently of dosage strength. The data does not have adequate information to determine the appropriateness of the dosage, however we included clinical indications for dosage reduction as candidate predictors in the model. Since, these variables were not selected for inclusion in the final model, it may be reasonable to assume that they do not an impact on the risk of bleeding or physicians are prescribing appropriate doses to patients. Fifth, caution should be taken when applying this score to those using apixaban. Due to the small number of events among apixaban users, the model may not be well optimized for use in this group. Sixth, we considered modifiable and non-modifiable predictors. Future work should evaluate the impact of modifying bleeding risk factors on the rates of bleeding in anticoagulated AF patients. Seventh, our exclusion of all patients with mitral valve disorders decreases the sensitivity of the non-valvular AF definition, by possibly excluding true cases of patients with non-valvular AF. Due to the nature of administrative databases, we lack information on clinical indicators, such as results from an echocardiogram, which could differentiate between types of valvular diseases that should be considered when diagnosing patients with non-valvular AF. Given the lack of information to characterize the type and severity of valvular disease and our desire to increase the specificity of the non-valvular AF definition, we excluded all patients with any diagnostic code for mitral valve disorders, at the expense of leaving out some patients that would be diagnosed as having non-valvular AF in the clinical setting. Finally, the model may not be generalizable to populations other than those used to derive and validate the model (e.g., the uninsured population). Specifically, the model only reflects the risk of bleeding in patients who are managed similarly to individuals in these databases and for those whom anticoagulants are considered safe to use. It will be important to evaluate prospectively the ability of the proposed predictive model to take into account potential changes in the quality of anticoagulation management by clinicians. Since our model performed well in two independent databases, we assume that it will performs well in a general population of insured AF patients. Of note, however, many of these limitations are shared by existing bleeding prediction tools.

In conclusion, the ABS provides clinicians with an easily accessible, practical tool for assessing a patient’s individual risk of bleeding for each anticoagulant. Prior schemes were limited in that they only provided a binary decision of whether or not to initiate anticoagulation therapy. The ABS facilitates the risk-benefit analysis of anticoagulation initiation by providing clinicians with additional information that allows for the quantification of bleeding risk by specific oral anticoagulant therapies.

## Supporting information

S1 Table ICD-9-CM codes used to define comorbidities. (DOCX)

S2 Table Models for bleeding prediction in atrial fibrillation patients receiving anticoagulation. (DOCX)

S3 Table Percentage of bootstrap samples in which a specific variable was selected to the oral anticoagulant-specific final model, MarketScan, 2007–2014. (DOCX)

S4 Table Model discrimination in the validation dataset for each anticoagulant. (DOCX)

S5 Table Model discrimination for age ≥75 in the derivation and validation datasets. (DOCX)

S1 Fig Calibration graph for model with interaction. Calibration curve relating observed and predicted bleeding rates across deciles of risk in A. Derivation Cohort (MarketScan) B. Validation Cohort (Optum Clinformatics). The 45 degree dashed line indicates perfect.
(TIF)

S2 Fig Calibration graph for model limited to individuals ≥75 years. Calibration curve relating observed and predicted bleeding rates across deciles of risk in A. Derivation Cohort (MarketScan) B. Validation Cohort (Optum Clinformatics). The 45 degree dashed line indicates perfect fit.
(TIFF)

S1 File 1-year risk of major hemorrhage hospitalization. Excel-based calculator that computes 1-year risk estimates of major hemorrhage hospitalization for each anticoagulant.
(XLSX)
