# Supplementary material for: A new model to predict major bleeding in patients with atrial fibrillation using warfarin or direct oral anticoagulants
Source: PLoS One. 2018 Sep 10;13(9):e0203599. doi: 10.1371/journal.pone.0203599 (PMC6130859; doi:10.1371/journal.pone.0203599)
Supplement: S1 Table — (DOCX) [file pone.0203599.s001.docx]

| **Condition** | **ICD-9-CM codes** |
| --- | --- |
| Alcoholism | 265.2, 291.1, 291.2, 291.3, 291.5, 291.6, 291.7, 291.8, 291.9, 303.0, 303.9,  305.0, 357.5, 425.5, 535.3, 571.0, 571.1, 571.2, 571.3, 980, V11.3 |
| Anemia | 280, 281, 282, 283, 284, 285 |
| Cancer | 140-172, 174-195, 200-208, 238.6 |
| Chronic pulmonary disease | 490-505, 416.8, 416.9, 506.4, 508.1, 508.8 |
| Coagulopathy | 286, 287.1, 287.3, 287.4, 287.5 |
| Coronary artery disease | 414 |
| Dementia | 290 |
| Depression | 296.2, 296.3, 296.5, 300.4, 309, 311 |
| Diabetes | 250 |
| Gastrointestinal bleeding | 455.2, 455.5, 455.8, 456.0, 456.20, 530.7, 530.82, 531.0, 531.2, 531.4, 531.6, 532.0,  532.2, 532.4, 532.6, 533.0, 533.2, 533.4, 533.6, 534.0, 534.2, 534.4, 534.6, 535.01,  535.11, 535.21, 535.31, 535.41, 535.51, 535.61, 537.83, 562.02, 562.03, 562.12,  562.13, 568.81, 569.3, 569.85, 578.0, 578.1, 578.9 |
| Heart failure | 398.91, 402.01, 402.11, 402.91, 404.01, 404.03, 404.11, 404.13, 404.91,  404.93, 425.4, 425.9, 428 |
| Hypertension | 401, 402, 403, 404, 405 |
| Intracranial bleeding * | **430**, **431**, 432, 852 |
| Ischemic stroke | 433, 434, 435, 436, 437, 438 |
| Kidney disease | 403.01, 403.11, 403.91, 404.02, 404.03, 404.12, 404.13, 404.92, 404.93, 582, 583.0,  583.1, 583.2, 583.3, 583.4, 583.5, 583.6, 583.7, 585, 586, 588.0, V42.0, V45.1, V56 |
| Liver disease | 070.22, 070.23, 070.32, 070.33, 070.44, 070.54, 070.6, 070.9, 456.0, 456.1, 456.2, 570,  571, 572.2, 572.3, 572.4, 572.5, 572.6, 572.7, 572.8, 573.3, 573.4, 573.8, 573.9, V42.7 |
| Myocardial infarction | 410, 412 |
| Other bleeding | 423.0, 459.0, 568.81, 593.81, 599.7, 623.8, 626.6, 719.1, 784.7, 784.8, 786.3 |
| Peripheral artery disease | 440.0, 440.2, 440.9, 443.9 |

# S1 Table. ICD-9-CM codes used to define comorbidities

* Bolded codes were used to define the outcome to increase specificity, while additional codes were used to increase the sensitivity as a predictor variable.
