# Supplementary material for: A new model to predict major bleeding in patients with atrial fibrillation using warfarin or direct oral anticoagulants
Source: PLoS One. 2018 Sep 10;13(9):e0203599. doi: 10.1371/journal.pone.0203599 (PMC6130859; doi:10.1371/journal.pone.0203599)
Supplement: S2 Table — (DOCX) [file pone.0203599.s002.docx]

# S2 Table. Models for bleeding prediction in atrial fibrillation patients receiving anticoagulation

| **Risk Score** | **Our Model** | **HEMMOR_2_HAGES** | **HAS-BLED** | **ATRIA** | **ORBIT** |
| --- | --- | --- | --- | --- | --- |
| *Variables* | Age | Age | Age | Age | Age |
|  | Kidney disease | Hepatic/renal disease | Abnormal renal/liver function | Renal disease | Abnormal kidney function |
|  | Prior bleeding | Hypertension | Hypertension | Hypertension | Prior bleeding |
|  | Ischemic Stroke | Prior bleeding | Prior bleeding | Prior bleeding | Anemia |
|  | Anemia | Stroke | Stroke | Anemia | Antiplatelet use |
|  | History of Cancer | Alcohol abuse | Drugs/Alcohol |  |  |
|  | Antiplatelet use | Anemia | Labile INR |  |  |
|  | Antiarrhythmic use | Cancer |  |  |  |
|  | Chronic pulmonary disease | Reduced platelet count/function |  |  |  |
|  | Heart failure | Genetic factors |  |  |  |
|  | Coronary artery disease | Fall risk |  |  |  |
|  | Diuretics use |  |  |  |  |
|  | Diabetes mellitus |  |  |  |  |
|  | Male sex |  |  |  |  |
|  | DOAC (vs. warfarin) |  |  |  |  |
|  | Dabigatran |  |  |  |  |
|  | Rivaroxaban |  |  |  |  |
|  | Apixaban |  |  |  |  |

Shaded variables were not available in either the derivation (MarketScan) or validation (Optum Clinformatics) datasets
