# Supplementary material for: A new model to predict major bleeding in patients with atrial fibrillation using warfarin or direct oral anticoagulants
Source: PLoS One. 2018 Sep 10;13(9):e0203599. doi: 10.1371/journal.pone.0203599 (PMC6130859; doi:10.1371/journal.pone.0203599)
Supplement: S3 Table — (DOCX) [file pone.0203599.s003.docx]

| Final Model Variables | Warfarin | Dabigatran | Rivaroxaban | Apixaban |
| --- | --- | --- | --- | --- |
| Age | **100** | **100** | **100** | 59.3 |
| Kidney disease | **100** | 6.0 | 8.6 | 6.2 |
| Prior bleeding | **100** | 8.2 | 6.9 | 36.4 |
| Ischemic Stroke | **91.4** | 27.9 | **78.1** | 10.1 |
| Anemia | **100** | **93.5** | 6.4 | 49.4 |
| History of Cancer | **93.5** | **62.5** | 30.4 | 9.2 |
| Antiplatelet use | **99.9** | 34.5 | 51.3 | 20.4 |
| Antiarrhythmic use | **92.3** | 32.9 | 13.1 | 6.0 |
| Chronic pulmonary disease | **100** | 6.6 | 7.0 | 49.8 |
| Heart failure | **99.9** | **77.6** | **60.7** | 56.4 |
| Coronary artery disease | 58.8 | **71.7** | 28.4 | 35.7 |
| Diuretics use | **99.2** | 18.4 | 23.6 | 8.3 |
| Diabetes mellitus | **98.9** | 24.2 | 9.1 | 14.6 |
| Male sex | 9.9 | 5.2 | **77.6** | 6.9 |

**S3 Table.** **Percentage of bootstrap samples in which a specific variable was selected to the oral anticoagulant-specific final model, MarketScan, 2007-2014**

Numbers correspond to percentages.

Bolded values corresponds to a given variable being selected in ≥ 60% of the bootstrap samples in the respective drug-specific model.
