# Supplementary material for: A new model to predict major bleeding in patients with atrial fibrillation using warfarin or direct oral anticoagulants
Source: PLoS One. 2018 Sep 10;13(9):e0203599. doi: 10.1371/journal.pone.0203599 (PMC6130859; doi:10.1371/journal.pone.0203599)
Supplement: S4 Table — (DOCX) [file pone.0203599.s004.docx]

| Anticoagulant | Validation cohort  (Optum Clinformatics) |
| --- | --- |
| Warfarin | 0.67 (0.65, 0.68) |
| Dabigatran | 0.72 (0.69, 0.76) |
| Rivaroxaban | 0.70 (0.68, 0.73) |
| Apixaban | 0.72 (0.67, 0.77) |

**S4 Table.** **Model discrimination in the validation dataset for each anticoagulant**
