# Supplementary material for: A new model to predict major bleeding in patients with atrial fibrillation using warfarin or direct oral anticoagulants
Source: PLoS One. 2018 Sep 10;13(9):e0203599. doi: 10.1371/journal.pone.0203599 (PMC6130859; doi:10.1371/journal.pone.0203599)
Supplement: S5 Table — (DOCX) [file pone.0203599.s005.docx]

| *Score* | Derivation cohort  (MarketScan) | Validation cohort  (Optum Clinformatics) |
| --- | --- | --- |
| Age ≥ 75 |  |  |
| Anticoagulation-specific  Bleeding Score | 0.63 (0.61, 0.64) | 0.63 (0.62, 0.65) |
| HAS-BLED score | 0.59 (0.58, 0.60) | 0.58 (0.56, 0.59) |
| ATRIA score | 0.59 (0.58, 0.61) | 0.59 (0.58, 0.61) |
| HEMORR_2_HAGES score | 0.60 (0.58, 0.61) | 0.58 (0.57, 0.60) |
| ORBIT score | 0.60 (0.58, 0.62) | 0.60 (0.58, 0.61) |

**S5 Table.** **Model discrimination [c-statistic (95% confidence interval)] for age ≥75 in the derivation and validation cohorts**
