# Supplementary figures and images for: A new model to predict major bleeding in patients with atrial fibrillation using warfarin or direct oral anticoagulants
Source: PLoS One. 2018 Sep 10;13(9):e0203599. doi: 10.1371/journal.pone.0203599 (PMC6130859; doi:10.1371/journal.pone.0203599)

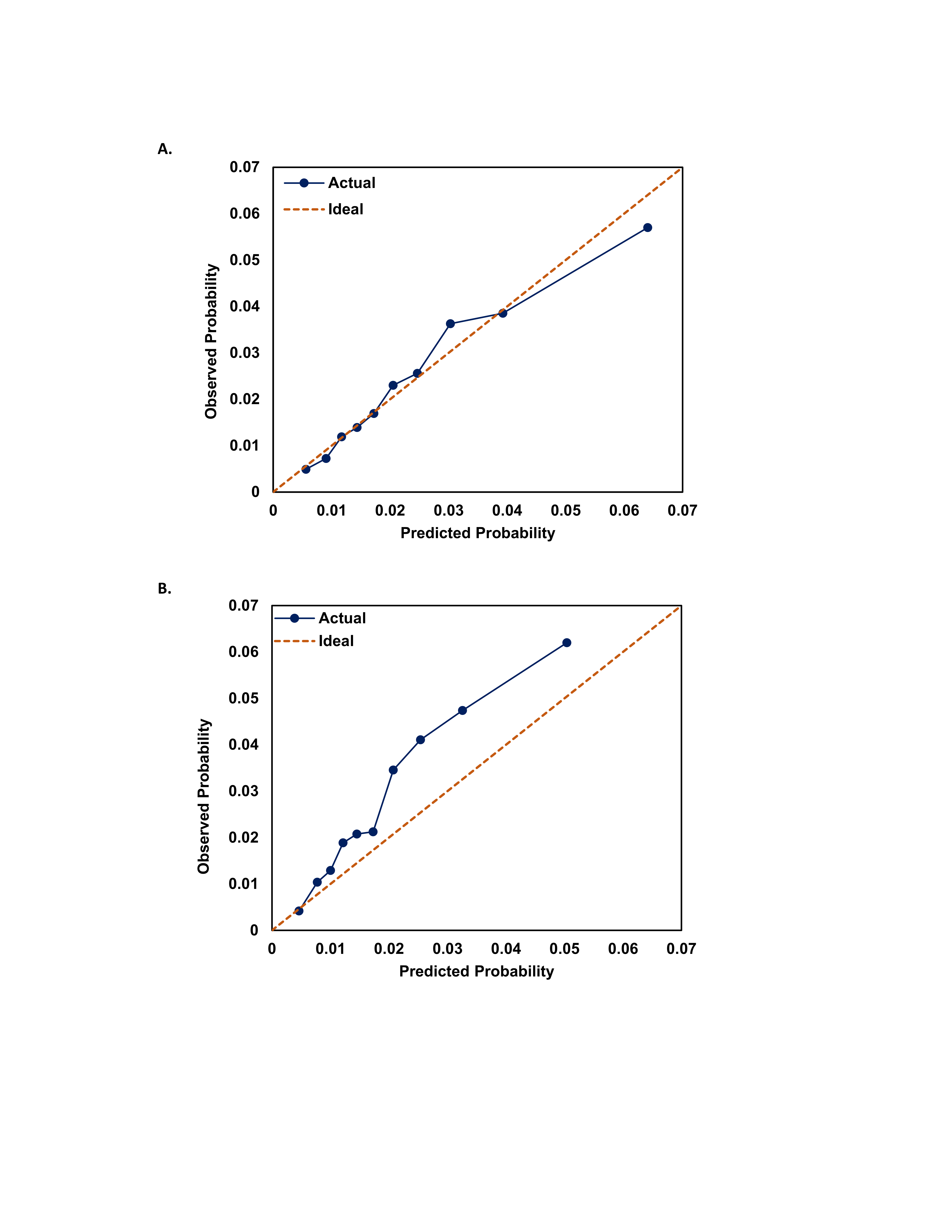

Supplement: S1 Fig — Calibration curve relating observed and predicted bleeding rates across deciles of risk in A. Derivation Cohort (MarketScan) B. Validation Cohort (Optum Clinformatics). The 45 degree dashed line indicates perfect. (TIF) [file pone.0203599.s006.tif]

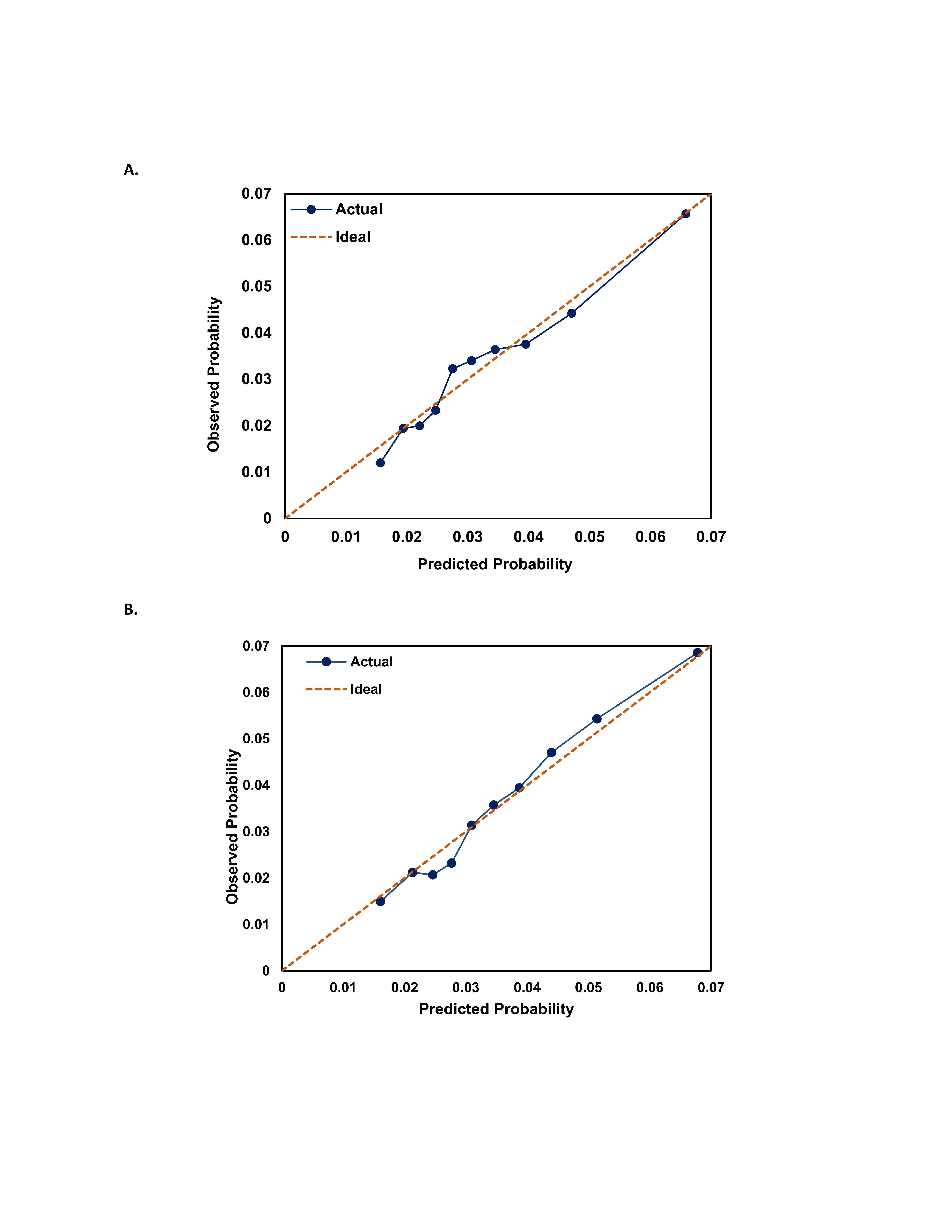

Supplement: S2 Fig — Calibration curve relating observed and predicted bleeding rates across deciles of risk in A. Derivation Cohort (MarketScan) B. Validation Cohort (Optum Clinformatics). The 45 degree dashed line indicates perfect fit. (TIFF) [file pone.0203599.s007.tiff]
